# Closer to assemble: Reply to “far from dismantled” by Root-Bernstein et al., 2023

**DOI:** 10.1016/j.heliyon.2024.e26705

**Published:** 2024-03-12

**Authors:** Nicolás Velasco

**Affiliations:** a Charles Darwin Research Station, Charles Darwin Foundation, Santa Cruz, Galápagos, Ecuador; b Departamento de Ciencias Ecológicas, Instituto de Ecología y Biodiversidad, Facultad de Ciencias, Universidad de Chile, Chile

We greatly appreciate the critical evaluation by Dr. Root-Bernstein and colleagues of our article. When we initially published our work, we anticipated such scrutiny, given their previous research highlighting the potential role of guanacos as dispersers of *Vachellia caven*. We view this scientific debate as an exciting opportunity to expand our understanding of this intriguing plant species.

First and foremost, we would like to express our gratitude for the authors' identification of an error in referencing [1]. As they correctly pointed out, this reference does not acknowledge that indigenous people extirpated guanacos from central Chile; instead, it was a process primarily occurring within the last five centuries [2]. While other sources do suggest that guanacos might have had limited populations due to the presence of a significant number of mountain lions in central Chile before European arrival [3], we concur that this information does not significantly contribute to the guanaco hypothesis section in our article. Therefore, we respectfully request the editor's assistance in amending our article to remove the sentence and reference from Ref. [1], to prevent any confusion among readers.

Having addressed this point, we will now systematically consider the other comments made by Root-Bernstein et al.

Our study emerged as a complementary endeavour following an exhaustive review of the Chilean matorral literature spanning the past half-century [4]. During this review, we identified two key issues with the development of the two proposed dispersal hypotheses for *V. caven* by Ovalle et al. (1990). First, the guanaco hypothesis was recurrently mentioned in the literature without substantial supporting evidence beyond superficial similarities to cattle. Second, the human-mediated hypothesis lacked adequate proof and was often dismissed due to uncertainties surrounding the labelling of “invasive” species for *V. caven*. Our primary objective in this study was to summarize the existing evidence for both contrasting dispersal hypotheses comprehensively. These topics are scattered across the literature, with limited formal evaluation. Our study highlights where these hypotheses can be located and offers an extensive list of available sources for further information. Nevertheless, as the authors correctly point out, little direct empirical evidence exists for either hypothesis. When additional studies become available, a follow-up study employing more concise methodologies, such as meta-analysis, will become essential.

The authors employ a famous phrase to conclude their discussion of the fossil record, “*lack of evidence for something is not evidence for anything”.* However, the authors inadvertently fell into a potential pitfall. In historical reconstructions, the absence of evidence can hold scientific significance, particularly in the context of animals and plants associated with humans [5]. We acknowledge the authors’ intention and concede that the wording of “*V. caven”* in the context of the fossil record section might be confusing. As indicated in the methods section, the focus extends to a broader taxonomic level. Rather than explicitly examining *V. caven*, readers should recognize that the presence of inadequate and unrelated fossil records in the western range (not limited to pollen) compared to the adequacy of records in the eastern range could imply that the species (or its relatives) were likely absent in the west, with minimal opportunities for vicariant events. Also, it is essential to note that the absence of fossil evidence should not be considered in isolation.

Regarding the speciation times presented, these values serve as the central results in both papers, making the inclusion of standard deviations or confidence intervals redundant for characterizing the precision of these estimates. It is self-evident that any time estimate will exhibit some degree of variation. In our paper, we merely emphasize that potential vicariance would be less suitable by the reduction in the time spans (from 10 My to 6 My). Additionally, in Bouchenak_Khelladi et al. [6], the speciation estimate pertains to *V. caven* and *V. farnesiana*, while the discordant findings of Gómez-Acevedo et al. (2010) involve the *V. farnesiana* node encompassing four species. Notably, *V. caven* falls as the last differentiating within this group, with estimated times even more recent than those presented in our manuscript (Gómez-Acevedo et al., 2010). The reference provided by the opponents, Garzione et al. [7], does not work correctly to sustain a vicariant event. This evidence suggests that paleoelevation reached up to 2000 m in the central Andean Plateau over the last 25 million years, with a consistent increase in the last 10 million years. While this is not the primary distribution area for the western range of *V. caven* and features different climates than the present era, it still implies that vicariance may be less plausible. Existing literature already indicates that the various *V. caven* populations inhabiting the western range rarely extend beyond premontane conditions [i.e., normally up-to 1000, rarely >2000 masl] ([8]; Sérsic et al., 2006)[9].

Root-Bernstein and colleagues have correctly pointed out that various species have successfully crossed the Andes, and we agree that this is not a matter of debate. However, their references encompass another range of mountains with a higher permeability, which may not adequately account for the substantial geomorphological variations across the Andes [10]. These references primarily pertain to the Tropical Northern Andes (TNA) rather than the central/south-central Andes (CSCA), with the former not exhibiting distinct climatic characteristics on both sides of the mountains, particularly in the current era [11] and to some extent during the Last Glacial Maximum (Willmes et al., 2017)[12]. Moreover, most of the TNA (except southern Ecuador) lacks the additional barrier mediated by the South American Arid Diagonal [13], or the ancient history of the Atacama Desert [14], which is critical for understanding the isolated nature of continental Chile.

The study cited by Root-Bernstein et al. concerning the Amotape—Huancabamba region straightforwardly attributes the presence of many plants on both sides of the barrier to similar climates and the lower altitude of the TNA in that specific region [15]. Furthermore, the authors referenced a study focused on Neotropical epiphytic orchids [16], which is not directly comparable due to the unique dispersal mechanisms within this group (as stated in the same study, “*dust-like* seeds”). Although Root-Bernstein et al. might contend on trait-neutrality, this orchid study relies on trait-driven explanations for species distribution and long-distance dispersal potential for their tiny seeds.

We infer that when authors mention *very long* periods and *long* distances, they seem to invoke a traditional, broad biogeographic view of long-distance dispersal (LDD). In our manuscript (end of section 3), we adhere to Jordano's (2018) *s.s* definition of LDD, which emphasizes the role of neighbouring genetic domain and not only distance in defining LDD. We used the terms “*probable*” and “*parsimonious*” not in the context of discussing long-distance dispersal but rather to consider the two main hypotheses. The authors fail to acknowledge that the primary purpose of the vicariance vs. long-distance dispersal discussion, as stated at the beginning of the corresponding section, is to address potential explanations for disjunct patterns. Although our article follows this structural approach, suitable for lay readers, the debate over these terms can be entirely avoided by focusing solely on the taxonomic pattern, which the authors conveniently sidestep. This taxonomic pattern, from the genus to a specific variety, effectively eliminates vicariance as a plausible explanation for the disjunct distribution. Moreover, it seems that the authors overlook evolutionary perspectives, such as the consistent morphology in the western range and considerable variability in the eastern range [8,[17], [18], [19], [20], [21]], which further supports the notion that the observed pattern is more likely a result of founder effects.

The authors base a significant portion of their LDD discussion on the concept of trait-neutrality and argue that discussions about probabilities are unproductive. While it is acknowledged that early literature has viewed LDD as trait-neutral, reducing LDD to random and improbable events can hinder biological perspectives. Arguing for trait neutrality treats species as interchangeable elements, and underestimates the importance of traits in ecological and evolutionary processes related to all types of dispersal [22,23]. In the case of LDD in plant species, several traits have been suggested as significant [24]. Moreover, when discussing probabilities in LDD, approaches based on mechanistic and kernel function models are used to estimate dispersal probabilities [22,25]. Arguing for trait-neutrality becomes irrelevant as these models benefit from the inclusion of propagule functional traits in both dispersal and establishment phases [26,27] and by incorporating dispersal agent traits [28]. Additionally, the reference cited by the authors to support trait-neutrality [29] pertains to the Neotropical Andean system, previously mentioned as different, and reduces dispersal traits to a single categorical variable, with most of the data coming from animal dispersal (supplementary material, [29]).

To gain a more comprehensive perspective on the potential role of humans as dispersers, authors should acknowledge the extensive literature supporting this notion in the CSCA (see e.g., the discussion in Ref. [30]). There is substantial historical evidence of human utilization of the region over the last millennia [31,32], with studies suggesting that Fabaceae species, such as *Prosopis* species, were not common in northern Chile, and human activities mediated their encroachment and presence in camelid diets [[33], [34], [35]]. Genetic diversity observed in several species’ populations in northern Chile (McRoostie et al., 2017; [36,37]) suggest introductions in the relatively recent past. Historical records also point to the trade of several Fabaceae species [38], even for some that never naturally occurred in Chile [39,40]. Another significant human use was as fuel [41], with the potential use of *Vachellia* in earth ovens dating back to the Archaic period in North America [42]. Several Fabaceae species in the CSCA are still used in medicine and rituals [43].

Utilizing the 165 km criterion to assess the potential for guanaco dispersal, while seeming unreasonable to Root-Bernstein et al., is well-founded and sufficiently challenging for our article. First, the authors claim that we did not consider the impact of land-use change. It is important to note that historical records from colonial times already indicate that *V. caven* formations were not as widespread as they are today. The encroachment of *espinales* occurred after the degradation of the *algarrobales* [44,45]. The distance mentioned between Los Andes and Mendoza was used to maintain consistency with the political borders of the two regions (West/Chile vs. East/Argentina). However, given the commerce and climatic similarities between both cities, a plausible interpretation is that the scattered trees found in Mendoza (Argentina) are a modern expansion from Los Andes city (Chile), possibly facilitated by historical cattle movements by train [46]. A more realistic eastern border around latitude ∼33°S might be San Luis in Argentina (for supporting data, refer to *V. caven* records on GBIF/iNaturalist). Secondly, our article has already considered that both ranges of *V. caven* were likely narrower during the Pleistocene. Recently, it has been predicted that *V. caven'*s eastern range was more distant than it is today, specifically at the same variety level (var. *caven*) (Velasco et al., 2023). This suggests the 165 km we used may have favoured the guanaco hypothesis, but more realistic interpretations would involve significantly larger separations between both ranges.

The authors raise two objections that lack solid support. First, they accuse us of lacking knowledge and imagination when addressing species ecology and non-human dispersal. Our article provides a comprehensive perspective on the species across most South American distribution, not just the Chilean viewpoint. For example, we have, for the first time, reviewed the potential of hydrochory extensively, which could be crucial for most varieties around the Paraná River [8,21]. On the other hand, the authors move to the other extreme, proposing too imaginative scenarios involving half-guanaco, half-washing mechanisms. However, they overlook the fact that the dispersal units in the hydrochory mechanism are the floating pods, which would be inevitably destroyed in the half-half scenario (e.g., guanaco chewing), and that *V. caven* seeds tend to submerge, making the washing system less plausible. While some of their ideas may have been influenced by Altamirano [47], it is essential to note that his work was focused explicitly on *P. tamarugo*, a Chilean endemic species.

Regarding our somewhat misleading statement about *V. caven* growth in areas with reduced competition due to perturbation, we acknowledge that there was no full clarity regarding which range we were referring to. This statement primarily pertains to the scenario in the eastern range, where the ecology of the species has been less studied. Therefore, the references provided by the authors for this well-known fact in the Chilean system are unnecessary.

We understand that our framing of the *Prosopis* spp. (now Neltuma and Strombocarpa) in our study may be challenging to grasp as it involves several interconnected ideas. Let's attempt to summarize it while incorporating the new references provided. First, we should approach Burkart's [48] reference with caution. He suggests that the palatability of *Prosopis* spp. may have influenced their distribution, but this likely occurred a long time ago and infrequently, resulting in the formation of endemic species in Chile (3 out of 7 species) before the Andes became a dispersal barrier. Second, *Prosopis* spp. shared between ranges should be reconsidered in light of the new evidence. As mentioned earlier, several studies indicate that humans may have introduced these species, at least in northern Chile.

We recognize the need for more knowledge about guanacos. Still, the evidence we have found is sufficient to suggest the lack of plausibility of guanacos (or other animals) as potential trans-Andean dispersers. Yet, not necessarily for dispersers *per se*. We also note that the evidence provided by the authors regarding guanacos’ broader feeding behaviour primarily comes from Patagonian animals. In contrast, Andean natural populations in north-central Chile have been proposed as specialized grazers [49]. While [50] shows that guanacos in north Patagonia feed on some shrubs, populations close to Mendoza city, which can be considered more similar to central Chile, tend to avoid shrubs year-round [51]. In fact, the study suggests that in premontane communities, where plant species richness tends to increase, guanacos focus more on specialization, mainly feeding on grasses. Ultimately, it is up to the authors to decide how to interpret these contrasting pieces of evidence.

We acknowledge that the new visual evidence provided by the authors supports the idea of guanacos being potential dispersers of *V. caven*. However, while our article recognised their role as plausible on local and regional scales, the data remains somewhat weak to conclude that they were potential trans-Andean dispersal agents. To clarify our perspective further and to counter the megafauna idea suggested by the authors, we can present a simple exercise.

In our article, we frequently used the example that flora from the eastern range did not cross the Andes (e.g., *Vachellia*), but similar examples can be used in the other direction. Considering the discussion (trait-neutrality, animals vs. human plausibility as trans-Andean dispersers), it would arguably be expected that there should not be a clear pattern in the distribution of a specific group. The latest Catalogue of the Chilean Flora [52] provides valuable information. For instance, let's focus on Chilean Fabaceae, specifically the native Phanerophytes. Nativeness implies that they are shared with another country, and phanerophytes pertain to shrubs or trees, which are under our scope. To encompass the species with a potential disjunct pattern between the Andes, we should exclude those also found in the Aysén and Magallanes regions since the low altitude of the Andes in those areas implies a minimal barrier effect. Among 296 species, 37 fall under this classification. Most of the shared species with Argentina have high-altitude tolerances (>3000 or even 4000 m above sea level) and start to inhabit the western range on premontane conditions, with minimal altitudes ranging from 1500 to 3000 m, indicating that cold tolerance is a must for being present at both sides. Guanacos appear to be suitable dispersers for all these species inhabiting *the Andes*. For instance, for the broader Chilean Fabaceae group, *Adesmia* spp., there is evidence of guanacos feeding on them in the Andes (Córtes et al., 2003). But species *not inhabiting the Andes* are not shared with Argentina for the same genus, even though they exhibit similar distribution ranges as *V. caven*. Moreover, in our Fabaceae exercise, most species that do not formally inhabit the Andes have a background of historical anthropogenic use (e.g., *Prosopis* spp., *Geoffrea*).

To begin our conclusions, we must address a couple of minor points. Firstly, we acknowledge that our study did not delve into discrete probabilities. Without getting lost in semantics, we concur with the authors that using terms like “plausible” would have been less perplexing. Secondly, and of greater significance, we agree that the questions surrounding the origin of *V. caven* remain largely unresolved. However, the evidence strongly suggests (as previously stated in our article) that animal vectors would have difficulty overcoming their limitations without humans orchestrating their movement and feeding dynamics. Our study encourages readers to discuss these ideas rather than sweep them under the rug. We advocate for further studies on the morphology and genetics of this intriguing tree, as suggested by the authors, to potentially track multiple dispersal events. In summary, we thank Root-Bernstein et al. for expanding this discussion to enrich our understanding of *V. caven*, and hopefully get closer to assembling its history.

A critical point of discussion which warrants more attention from readers is our sincere agreement with the importance of *V. caven* underscored by Root-Bernstein et al. Our team does not advocate reclassifying the species, as the authors imply. In fact, in our article's synthesis, we explicitly stated that “*V. caven should continue to be treated as native*.” Archaeophytes are also considered native in their recipient areas. The discussion we have clarified here would similarly apply to the northern Chilean populations of *Prosopis* spp., but we believe that no one would consider reclassifying these species; simply, that is not something open to debate.

Lastly, when we discuss *V. caven* resembling an invasive species, we are not making a naïve declaration. The authors may have fallen into the trap of judging species solely by their origin and viewing introductions as a threat (Davis et al., 2014)[53]. However, we intend to draw attention to the demographic and historical context that the species may have had, not just its impact [54]. Specifically, we have recently published a study that assesses the facilitative effects of *V. caven* throughout most of its distribution, from Chile to Paraguay [55]. Bearing all of this in mind, we anticipate the following: if you consider the extensive *V. caven* positive effects, in addition to its potential introduction, it becomes necessary to reconsider the positive implications that historical “invasions” may have had and the role of humans in shaping biodiversity. We believe that is the most promising aspect of our research.

## CRediT authorship contribution statement

**Nicolás Velasco:** Conceptualization, Investigation, Writing – original draft, Writing – review & editing.

## Declaration of competing interest

The authors declare that they have no known competing financial interests or personal relationships that could have appeared to influence the work reported in this paper.
